# Issues of informed consent for intrapartum trials: a suggested consent pathway from the experience of the Release trial [ISRCTN13204258]

**DOI:** 10.1186/1745-6215-7-13

**Published:** 2006-05-11

**Authors:** Gillian Vernon, Zarko Alfirevic, Andrew Weeks

**Affiliations:** 1 Clinical Trial Administrator, School of Reproductive and Developmental Medicine University of Liverpool, Liverpool, UK; 2 Professor of Fetal & Maternal Medicine School of Reproductive and Developmental Medicine University of Liverpool, Liverpool, UK; 3 Senior Lecturer in Obstetrics and Gynaecology School of Reproductive and Developmental Medicine University of Liverpool, Liverpool, UK

## Abstract

Service users within the NHS are increasingly being asked to participate in clinical research. In Liverpool Women's NHS Foundation Trust, approximately 35% of women take part in research during their pregnancy. For many studies the consent process is simple; information is provided and signed consent is given. There is a difficulty, however, with obtaining informed consent from women in pregnancy who become eligible only when they develop unforeseen complications, especially when they occur acutely. The problem is compounded with women in labour who may be frightened, vulnerable, in pain, under the effect of opiate analgesia, or all of the above. If research to improve the care of these women is to continue, then special consent procedures are needed. These procedures must ensure that the woman's autonomy is protected whilst recognising that women under these circumstances vary enormously, both in their desire for information and their ability to comprehend it. This paper will discuss the obtaining of consent in this situation, and describe an information and consent pathway for intrapartum research which has been developed in collaboration with consumer groups as a way in which these issues can be tackled.

## Introduction

Ethical issues concerning research on human subjects have been covered extensively and there are a number of guidelines published to provide authoritative advice for the research community [1-5]. Such guidance usually states that informed consent must be gained from the trial participants before they can be recruited to the trial. Ideally, several days should be allowed for individuals to consider the research, and their possible participation in it, giving them a chance to discuss it with their families, friends and family doctor [4]. The patient's consent is then initially confirmed with a signature, although it should be made clear that the agreement is not binding.

It is widely acknowledged that there are vulnerable groups from whom gaining consent for clinical research presents ethical difficulties. Such groups include children, young people and adults who 'lack capacity', for example those with mental illness or learning difficulties. Specialised guidance concerning gaining consent from these specific groups is available [3,7]. One vulnerable group, however, which appears to have been forgotten, is labouring women. Guidelines addressing the recruitment of women into intrapartum clinical trials are not yet readily available from the professional organisations, although 3 consumer groups [Association for Improvements in the Midwifery Services (AIMS), National Childbirth Trust (NCT) and the Maternity Alliance] have gone some way in addressing this need with the publication of their 'Charter for Ethical research in Maternity Care' in 1997 [6].

The Release trial is an example of an ongoing intrapartum randomised controlled trial for which a trial-specific information and consent pathway was developed in collaboration with consumer groups. This paper describes this pathway and discusses its possible adaptation for future intrapartum research. This is particularly important as there are fears that in attempting to safeguard the wellbeing of trial participants, consent procedures could prevent intrapartum research. Furthermore, studies suggest that women value the opportunity to take part in research [8,9], and from a medical perspective it is clear that good quality research is crucial if the quality of intrapartum care is to improve.

## The Release trial

The Release trial is an intrapartum randomised-controlled trial of umbilical oxytocin injection for the treatment of retained placenta. Retained placenta occurs after 2% of deliveries and is diagnosed when the placenta fails to deliver 30 minutes after childbirth. Due to its acute clinical context it has thrown up a number of ethical issues concerning informed consent. The consent pathway for the trial was developed in consultation with local and national consumer groups, local experts in the field of consent issues and the London Multi-centre Research and Ethics Committee (MREC). The issues faced during this process are detailed below, with examples of how the Release trial addressed them and points for consideration by other researchers.

### Antenatal information

The provision of sufficient accurate information is an essential part of seeking informed consent. Indeed, when seeking consent the quality and clarity of the information given should be the paramount consideration [3]. For intrapartum trials it is not possible to fulfil all of the criteria above, especially that of time. Hence, there has been a move to provide information to all pregnant women antenatally, despite the fact that only a small number will be eligible to participate in a study. However, there is a conflict between promoting 'normality' in pregnancy and labour, and routinely presenting women with research information. For many women the risk of experiencing these adverse events is small and to present women with a detailed discussion of each complication's symptoms and management risks, unnecessarily detracts from labour as being a normal physiological process.

To date there have been no studies specifically designed to evaluate women's antenatal information requirements concerning obstetric complications and associated research. However, a study by Jackson and colleagues in Canada studied women's desire for information about risks associated with epidural analgesia [10]. The researchers used a questionnaire covering demographic data, epidural and consent information. Fifty six women who had requested an epidural were asked to give a score between 0 and 10 for the question 'risks should be discussed prior to labour'. The average score to this question was 8/10 (0 = least, 10 = most). The authors concluded that labouring women want to hear about all potential epidural complications, and for risks to be explained to them before labour. Although this study addressed information regarding risks associated with a treatment, specifically epidural analgesia, it may suggest that some women wish to be informed of potential risks associated with intrapartum research before they go into labour.

A prospective study of women's views by Lavender and colleagues explored aspects of childbirth that women perceived as being important and as contributing to a positive birth experience [9]. Women considered information to be an important factor contributing to a positive labour experience, with 154 (37%) feeling unprepared for labour because of either a lack of information or their own unrealistic expectations. Some women stated that they wished they had had more information antenatally. Those women who felt prepared, and who felt they had acquired adequate and accurate information were less likely to view their labour negatively. The nature of the information women would regard as adequate, or that they would like to receive antenatally requires further exploration.

**Figure 1:**
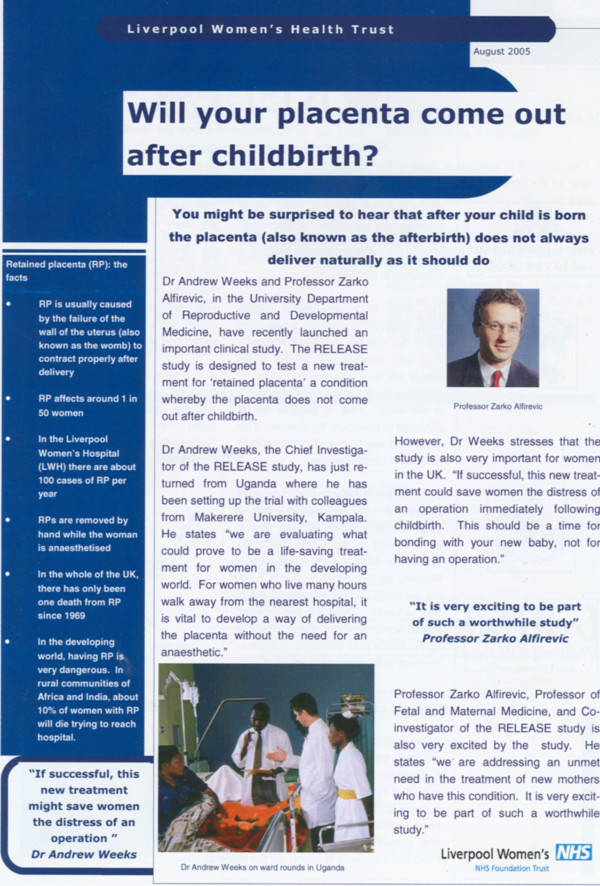
Front cover of the Release study newsletter.

For the Release trial antenatal information is provided through a variety of means. First, brief information is provided to women at the 'booking' visit at 9–14 weeks, in the form of an A4 information sheet in their case notes. This information provides an introduction to the study – what it is called, why it is being conducted, a brief overview of the technique being evaluated, and who to contact for further information. Additional information in the form of a 4-page tabloid-style brochure has also been distributed in antenatal clinics and is available on labour wards in the UK trial sites (figure 1). The purpose of this is to provide further details of who the study is organised by and how it is funded. It also provides further details of the technique being evaluated, advice on how to take part, contact details and pictures of the research team so as to develop familiarity. These sources of information are backed up with posters in the antenatal clinics and publicity in the local press.

All the above publicity is provided to raise awareness of the trial antenatally and give women the option of obtaining more information if they wish.

### Web-based information

For each individual the requirement for information is different depending on their personality, time pressures, and interest. Some women will wish to know in advance every risk associated with pregnancy and the research being conducted to address those adverse events, while others may not wish to know about such risks prior to them arising.

A good medium to use for addressing differing information requirements is the internet. As a source of information, it allows a person to obtain as little or as much information as they wish, and to retrieve that in which they are interested.

**Figure 2:**
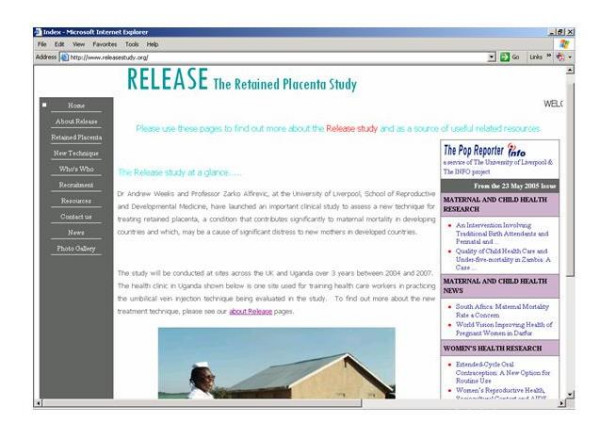
Release study website homepage .

In all of the Release study documentation available to women, reference is made to the study website to which women may turn to address their different information needs (figure 2). The website provides information of a varying nature; non-clinical and clinical, plus latest news items and downloadable resources including presentations and trial documents. It therefore provides a good source of information addressing questions of a various nature and level. However, there are limitations associated with the dissemination of research information via the internet, for example not all potential participants will have access to computers and, for smaller studies, the expense of setting up and maintaining a website may be prohibitive.

### Information at the time of recruitment

Finally, there is the controversial issue of seeking consent while a woman is in labour. It may be argued that some women are unable to give their full attention to the details of a research study and to think carefully about the implications of becoming involved in the research while they are in labour. During this time women may be experiencing painful contractions, drowsy through the effects of opioids and anxious about the possibility of complications. In addition, many women may feel vulnerable. They may therefore be afraid of not complying with their carer's suggestions and may feel pressurised into giving their consent to participate in clinical research. As such, it is possible that a woman's competence to give informed consent during labour may be compromised [11].

In some trials of emergency treatments outside labour, researchers have sought retrospective consent [12,13]. Whilst this may be suitable for trials in which the time period between diagnosis and need for randomisation is very short or the patient is clearly incapacitated, being in labour is not in itself an indication for doing this. Jackson and colleagues found that women in labour were as able to give informed consent as are other members of the patient population [10]. The investigators surveyed 60 women who were actively in labour without epidural analgesia, to assess women's ability to understand epidural risks during this time. They found that the women wanted to know about labour epidural analgesia and have all risks disclosed. The authors concluded that no anticipated variable (i.e. labour pain, anxiety, opioid pre-medication, duration of labour pain, desire for an epidural, previous epidural experience or level of education or age) correlated with a woman's ability to understand epidural risks, and that all of the women had at least a moderate understanding of the risks during active labour. A further study investigated the ability of women in labour to recall the risks of epidural, previously explained to them in labour [14]. This study also questions the assumption that women in labour lack the capacity to give informed consent. The recall of risks by women in labour was found to be similar to that of other patient groups, and did not appear to be affected by parity or the reported level of pain.

So if women can comprehend the research despite the stress of labour, do they feel pressurised to participate due to their vulnerable state? A study by Dorantes and colleagues, looking at the factors that influence women's decisions to participate in obstetric anaesthesia research, suggested that the environment, in which consent for obstetric studies was sought, was not coercive [15]. In this study, only one woman out of 166 consenters reported feeling pressurised to consent.

**Figure 3:**
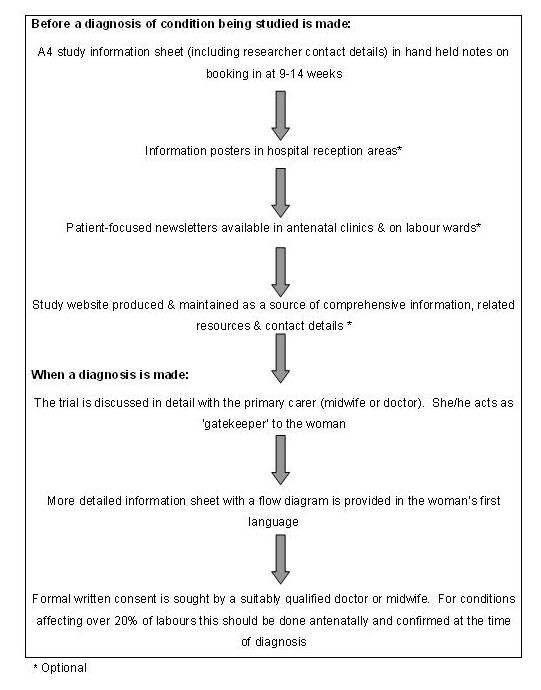
Consent pathway for intrapartum research.

For the Release trial, these issues were addressed by consultation with consumer groups who provided valuable input from women's perspectives, addressing questions raised by the ethics committees during the process of protocol development. The result was a consent pathway (figure 3) drawn up by the University of Liverpool School of Reproductive and Developmental Medicine in collaboration with the North West Obstetrics and Gynaecology Clinical Trials Network (NWCTN), and representatives from local consumer groups.

When the diagnosis of retained placenta is made, 30 minutes following delivery of the baby, the trial may be discussed by the researcher in further detail with her attending midwife or doctor to ascertain whether it is appropriate to approach her, considering her emotional and physical state. Whilst this would be considered good research practice anyway, this role should be made explicit, with the primary carer acting as gatekeeper on behalf of the labouring woman, preventing unwanted disturbance and acting as advocate for her. If appropriate, the woman will then be provided with a more detailed information sheet, which is also available in Somali, Arabic, and Luganda, and which contains a flow diagram to facilitate understanding. This information builds upon that the woman received from the various sources antenatally. At this point, the doctor or midwife seeking consent, who will be fully trained in consent procedures, would confirm some level of understanding on the woman's part, by encouraging her to reiterate important points of the study. Only following this will formal written consent be sought.

## Discussion

Evidence suggests that despite concerns of vulnerability, pain and opiate analgesia, women do have the capacity to give informed consent to take part in intrapartum studies. Furthermore, women are often willing to participate in research, although it is acknowledged that there are many contributing factors to their decision making processes, depending upon their individual circumstances and beliefs [16]. This capacity, willingness and complexity warrants the need for guidance in the process of gaining consent in intrapartum trials to ensure researchers seek consent according to good ethical principles. Current guidance developed by professionals in conjunction with individual consumers appears to display a lack of understanding of what consumers want, and struggles to strike a balance between providing adequate information without causing information overload and unnecessary anxiety. In this paper, we have described a pathway developed as one way to tackle these issues.

The information and consent pathway proposed here seeks to ensure that women have the opportunity to become fully informed. However, there is a choice issue which requires consideration when it comes to adhering to the fully informed consent process prescribed by ethics committees. When providing perinatal research information it is important to note that some women may prefer not to consider all of the information, and should perhaps be given the option to 'opt-out' of the fully informed consent process. Anecdotal evidence suggests that some women would prefer not to have to choose whether or not to participate in research, and would feel relieved if the onus was removed from them, indeed some women expressed their belief that research need not be mentioned and should be incorporated into routine care [16].

Up to now, the lack of coherent guidance has made it problematic for the research community to gain ethical approval for recruiting women into perinatal clinical trials. Currently, the onus is on individual researchers to argue their case to ethics committees on gaining ethical approval for perinatal studies based on individual research protocols. This is leading to inconsistent and often conflicting advice from various ethics and research bodies, concerning best practice in seeking consent from women participating in perinatal clinical trials. In turn, this may lead to confusion and deter research into perinatal adverse events.

Ethics and research bodies request that women be fully informed before consent is sought to participate in intrapartum trials. However, given the issues addressed in this paper, it is difficult to assess if a woman is indeed fully informed before seeking consent. A questionnaire survey of women who participated in the ORACLE trial (a trial of antibiotics in preterm labour) suggested that no matter how well we try to optimise the provision of information, it may not always be possible to demonstrate full understanding of trial purpose by participants [17]. Therefore emphasis should be placed on presenting information as fully as possible and having consumers involved in the writing and design process in an attempt to enhance understanding.

It is important therefore to consider to what extent we should negotiate. For example, if women do not receive adequate information antenatally either because of lack of access, or by choice (opting out of the fully informed consent process), should they automatically be excluded from research without giving them the choice to participate? Is this not unethical in itself?

These issues add to the concerns of the research community in general, regarding the increasingly complex procedures for gaining ethical approval to conduct clinical research. The process of gaining ethical approval in the UK has been described as 'bureaucratic' [18,19]. The concern is that important trials will be unnecessarily delayed, will incur additional costs without any increased protection for trial subjects and that UK partners may become unwelcome in international trials [18].

We are not proposing a standardised approach to seeking informed consent, but a framework with optional elements that can be adjusted to take into account the individuality of both the study and the women who are participating in it. Evidence suggests that women consider an individualised research approach towards them as important and that they want their individual situations to be acknowledged. If this is done then it is likely to encourage research participation [16]. Exactly how this can be achieved for a particular study is an area for further exploration by the researchers concerned.

From the experience of the Release trial it was important to involve consumer groups at an early stage of protocol development to consider how it was most appropriate and acceptable to women, to seek consent during third stage labour. It has also been important to consider the resource implications of developing, maintaining and staffing the elements of the agreed pathway throughout the duration of the trial. It is also apparent that compliance to the information and consent pathway may vary from site to site, and this needs to be monitored throughout the trial. Formal assessment of the Release consent process is ongoing within its principle site at Liverpool Women's NHS Foundation Trust. The authors will report their findings in due course as to whether the information and consent pathway they propose here has achieved the desired outcome in terms of the provision of appropriate information to help women make informed decisions about their participation in intrapartum research.

## Authors' contributions

GV carried out the literature search and writing of the manuscript.

AW provided the original idea for the manuscript and helped draft out the manuscript.

ZA revised the manuscript providing comments on its intellectual content.

All authors have given final approval of the version to be published.
